# Unexpected ICU Transfer and Mortality in COVID-19 Related to Hospital Volume

**DOI:** 10.5811/westjem.2022.8.57035

**Published:** 2022-11-01

**Authors:** Cassidy M. Dahn, Sana Maheshwari, Danielle Stansky, Silas Smith, David C. Lee

**Affiliations:** * NYU Grossman School of Medicine, Ronald O. Perelman Department of Emergency Medicine, Division of Critical Care, New York, New York; † NYU Grossman School of Medicine, Ronald O. Perelman Department of Emergency Medicine, New York, New York; ‡ NYU Grossman School of Medicine, Ronald O. Perelman Department of Emergency Medicine, Division of Medical Toxicology, New York, New York; § NYU Grossman School of Medicine, Ronald O. Perelman Department of Emergency Medicine, Department of Population Health, New York, New York

## Abstract

**Introduction:**

Coronavirus 2019 (COVID-19) illness continues to affect national and global hospital systems, with a particularly high burden to intensive care unit (ICU) beds and resources. It is critical to identify patients who initially do not require ICU resources but subsequently rapidly deteriorate. We investigated patient populations during COVID-19 at times of full or near-full (surge) and non-full (non-surge) hospital capacity to determine the effect on those who may need a higher level of care or deteriorate quickly, defined as requiring a transfer to ICU within 24 hours of admission to a non-ICU level of care, and to provide further knowledge on this high-risk group of patients.

**Methods:**

This was a retrospective cohort study of a single health system comprising four emergency departments and three tertiary hospitals in New York, NY, across two different time periods (during surge and non-surge inpatient volume times during the COVID-19 pandemic). We queried the electronic health record for all patients admitted to a non-ICU setting with unexpected ICU transfer (UIT) within 24 hours of admission. We then made a comparison between adult patients with confirmed coronavirus 2019 and without during surge and non-surge time periods.

**Results:**

During the surge period, there was a total of 86 UITs in a one-month period. Of those, 60 were COVID-19 positive patients who had a mortality rate of 63.3%, and 26 were COVID-19 negative with a 30.8 % mortality rate. During the non-surge period, there was a total of 112 UITs; of those, 24 were COVID-19 positive with a 37.5% mortality rate, and 90 were COVID-19 negative with a 11.1% mortality rate.

**Conclusion:**

During the surge, the mortality rate for both COVID-19 positive and COVID-19 negative patients experiencing an unexpected ICU transfer was significantly higher.

## INTRODUCTION

Coronavirus disease 2019 (COVID-19) is a global pandemic that continues to affect the United States. Since its nascence in China’s Wuhan province in late 2019, the outbreak has evolved with startling rapidity with more than 304 million confirmed cases and an estimated 5.4 million deaths globally.^1^ In many parts of the US, the dramatic spread of COVID-19 led to waves of patients that overwhelmed hospitals and healthcare systems. While data continues to accrue regarding risk factors for severity of illness and mortality, management, ventilation strategies, imaging, and diagnosis, much remains unknown.^2^ Specifically, there is limited literature published to date on COVID-19 patients with regard to unplanned intensive care unit (ICU) transfer (UIT). Literature to date is focused on risk prediction modeling and includes any ICU transfer, but it does not speak to hospital capacity and inpatient volume and its effect on UIT and does not focus on UIT within the first 24 hours.^3^–^7^

The risk of UIT is highest in the first 24 hours of admission with an incidence reported between 2–5% across hospital systems.^8^–^9^ Previous retrospective studies have found that patients admitted with respiratory conditions, sepsis, myocardial infarction, significant comorbidities, tachypnea, or abnormal lab findings are at greatest risk for UITs.^9^–^10^ While there is a lack of general consensus as to which factors are more predictive, UITs have consistently demonstrated an increase in morbidity and mortality in comparison to those patients directly admitted to the ICU from the emergency department (ED).^8^, ^11^–^12^

COVID-19 primarily affects the respiratory system with hypoxia and increased work of breathing and progression to acute respiratory distress syndrome (ARDS). These indicators have been associated with increased UIT and rapid decompensation in prior studies of initial variants.^9^–^10^, ^13^ During COVID-19, it was reported that 5–12% of patients required ICU level of care. Research to date on COVID-19 UITs report that 5–13% of patients required ICU upgrade after admission to the floor with a median time of transfer between 2.45–2.58 days.^3^–^4^ Identified predictive variables for COVID-19 ICU transfers include respiratory rate, white blood cell and lymphocyte count, oxygen saturation, and elevated C-reactive protein.^4^–^7^ For patients admitted to the hospital the overall mortality is around 4–5%, but for those requiring mechanical ventilation it is reported to be 23%.^3^ Given the severity of illness associated with COVID-19 and high rates of respiratory failure, hospital capacity and resources have been shown to be near or at capacity in many hospital systems. The effect that hospital capacity and volume may have on UIT in COVID-19 has minimal literature published to date.

## METHODS

### Study Design and Patients

This was a retrospective cohort study of a single health system comprising four EDs and three tertiary hospitals in New York, NY, from March 30–April 30, 2020 (at time of high inpatient and ICU census and near-full capacity defined as surge) and October 30, 2020–January 31, 2021 (at time of lower inpatient and ICU census and non–full capacity defined as non-surge) for UITs. Patients were selected for inclusion on the basis of a COVID-19 diagnosis confirmed by polymerase chain reaction testing; age ≥18 years, and admission from the ED to a non-ICU (and non-procedural) level of care, who required an upgrade to an ICU level of care (UIT) within 24 hours of admission. Exclusion criteria were pediatric patients, and patients who were admitted to hospice, directly to the ICU, or to the ICU post-procedure (ie, from the operating room, catheterization suite, interventional radiology, or endoscopy), as well as patients admitted or transferred from outside hospitals directly to the ICU.

**Population Health Research Capsule**

What do we already know about this issue?

*Unexpected intensive care unit (ICU) transfer (UIT) demonstrates an increase in morbidity and mortality compared to direct ICU admission. In COVID-19, incidence of UIT is 5–12%*.

What was the research question?

*We sought to determine the effect of hospital volume on patients with respect to UIT during the COVID-19 surge*.

What was the major finding of the study?

*During higher hospital volume, mortality was 1.7 (P = 0.02) and 2.7 (P = 0.01) times higher in COVID-19 positive and COVID-19 negative UITs, respectively*.

How does this improve population health?

*The effect of hospital capacity must be accounted for when assessing risk for UIT to optimize care and triage of critically ill patients*.

The study was approved by the institutional review board of the participating hospitals. Written consent was waived as there was no intervention nor risk to the subjects during the performance of this study.

### Data Collection

We abstracted data from the hospital electronic health record (EHR) (Epic Systems Corporation, Verona, WI). Some of the data for this subset of patients was obtained from a shared database and a prior publication in a larger institutional study.^14^ Demographic characteristics included age, self-identified gender and race/ethnicity, and language. We obtained comorbidities (hypertension (HTN), hyperlipidemia (HLD), diabetes, coronary artery disease (CAD), congestive heart failure (CHF), asthma, chronic obstructive pulmonary disease (COPD), cancer, cirrhosis, chronic kidney disease, end-stage renal disease (ESRD), immunocompromised status, body mass index (BMI), smoking status), and admission diagnoses. We reviewed ED oxygen requirements as well as outcomes from the patients’ inpatient course (need for intubation/mechanical ventilation, mechanical ventilation days, time from admission to ICU transfer, time from admission to death, ICU length of stay [LOS], hospital LOS, and mortality).

### Statistical Analysis

We performed a descriptive analysis to compare the demographic and clinical characteristics for the four groups of ICU upgrades analyzed in this study (ie, surge COVID-19 positive, surge COVID-19 negative, non-surge COVID-19 positive, and non-surge COVID-19 negative). Continuous variables were expressed as means and/or medians, and categorical variables were expressed as proportions. We analyzed continuous variables using ANOVA or Kruskal-Wallis equality-of-populations rank tests, as appropriate. Categorical variables were analyzed using chi-squared tests. For our primary outcome of mortality among the ICU upgrades, we used chi-squared tests to compare the proportion of patients who died among these subgroups. We specifically compared mortality among the COVID-19 positive ICU upgrades between the surge and non-surge periods and also among the COVID-19 negative ICU upgrades between the surge and non-surge periods. For these outcomes we used a Bonferroni adjusted *P*-value of 0.025 to account for the two comparisons performed. All statistical analyses were performed in Stata statistical software v16.2 (StataCorp, LLC, College Station, TX)

## RESULTS

### Demographics of the Study Population

The ICU upgrades had a median age of 65.5 years. We did not identify any statistically significant differences (*P* = 0.61) in the median age among the four groups of patients analyzed (ie, surge COVID-19 positive, surge COVID-19 negative, non-surge COVID-19 positive, and non-surge COVID-19 negative). Approximately 66% of ICU upgrades were male and, notably, there was a similar gender distribution among COVID-19 positive and COVID-19 negative ICU upgrades in the surge period, whereas there was a much higher proportion of male patients among COVID-19 positive ICU upgrades in the non-surge period at 88% when compared to 56% of COVID-19 negative ICU upgrades during the same period (*P* < 0.01). Patients who identified as non-White comprised 47.2% of the ICU upgrades. In the surge periods, the number of non-White patients was higher among COVID-19 positive ICU upgrades (65.0% COVID-19 positive and 34.6% COVID-19 negative), but was similar in the non-surge period (45.8% COVID-19 positive and 43.8% COVID-19 negative) (Table 1**)**.

### Comorbidities of the Study Population

Of the 12 comorbidities analyzed in our study, on average 61.6% of ICU upgrades had a history of HTN, 44.4% had HLD, 13.6% had asthma, 11.1% had COPD, 17.7% had cancer, 20.7% had CKD, 22.7% had CAD, 17.7% were immunocompromised, 0.5% had cirrhosis, 15.2% had CHF, and 7.1% had ESRD. When comparing the four groups of patients analyzed, we only found a statistically significant difference in immunocompromised status (*P* < 0.01). In the surge period, immunocompromised patients accounted for 15.0% of COVID-19 positive ICU upgrades vs 7.7% of COVID-19 negative related ICU upgrades, whereas in the non-surge period, immunocompromised patients accounted for 0.0% of the COVID-19 positive ICU upgrades vs 27.3% of the COVID-19 negative-related ICU upgrades. The median BMI of ICU upgrades was 27.9, and while we did not find a statistically significant difference among the median BMI among the four groups analyzed (*P* = 0.10), we did note that there was a statistically significant higher BMI among COVID-19 positive ICU upgrades at 29.0 compared to COVID-19 negative ICU upgrades at 27.0 when the surge and non-surge patients were combined (Table 1).

### Inpatient Outcomes

For all patients with UIT within 24 hours during surge (COVID-19 positive and negative) and non-surge (COVID-19 positive and negative) the average time from admission to transfer was 13.0, 10.3, 12.8, and 11.5 hours, respectively. A majority of the patients required mechanical ventilation (76.7%) in the surge COVID-19 positive population, followed by 50.0% in the non-surge COVID-19 positive population and only 19.2% and 15.9% in the surge and non-surge COVID-19 negative populations, respectively. COVID-19 positive patients in surge and non-surge times had overall longer mechanical ventilation (MV) days, ICU and hospital LOS (Table 2**)**.

### Mortality Among ICU Upgrades

During the surge time, there were 86 UITs over the course of a month. Sixty were COVID-19 positive patients who had a mortality rate of 63.3%; 27 were COVID-19 negative with a 30.8% mortality rate. In the subsequent non-surge period, 112 UITs occurred (37 per month). Twenty-four were COVID positive, with a 37.5% mortality rate; 90 were COVID negative, with an 11.1% mortality rate. During surge, mortality among COVID-19 positive patients was 1.7 times higher (*P* = 0.02) and 2.7 times higher among COVID-19 negative patients (*P* = 0.01) when compared to the non-surge period (Table 2).

## DISCUSSION

COVID-19 mortality has been devastating both nationally and internationally at much higher rates than seen in prior viral-related respiratory diseases. As the COVID-19 pandemic continues to press hard and affect so many communities, it is vital to identify risk factors for patients who will deteriorate or expire quickly, so that recognition, interventions, and appropriate levels of care can be accomplished. The effect of hospital capacity and volume must be accounted for as well.

In the setting of COVID-19, a lean, resource-strained, hospital system requires utmost efficiency as well as a quality-conscious healthcare practice that prevents unnecessary harms. It is important to follow guidelines and appropriately use the already limited ICU bed capacity. Hospitals can look to national ICU admission guidelines (eg, the Society of Critical Care Medicine and the American Association for the Surgery of Trauma guidelines, as well as disease-specific guidelines such as those for pneumonia from the American Thoracic Society). However, these guidelines are not always specific enough and do not consider different levels of organizational capabilities or patient-associated factors that can vary among hospitals.^15^–^17^

The purpose of this study was to examine UITs during the different times of inpatient and ICU capacity and assess the effect on a high-risk population of patients who were transferred to a higher level of care within 24 hours of admission. The correlations in and of themselves may also be of potential benefit to clinicians in terms of the assessment of COVID-19 patients who may be at risk for decompensation and may benefit from early recognition, potential intervention, or higher level of care. It will also be useful to evaluate whether patients admitted with COVID-19 who experience UIT within 24 hours similarly have increased morbidity and mortality as seen with other cohorts of patients with early UIT.

Not surprisingly, the vast majority (85%) of patients who died within 24 hours had high oxygenation demands while in the ED (defined as more than nasal cannula supplementation). Patients with respiratory disease have previously been identified as a higher risk pathology correlated with unexpected rapid deterioration identified by expiration or ICU transfer within a short time after admission. In the case of COVID-19, the sickest patients have been affected by the virus primarily by respiratory failure. Better differentiation of patients who may do well in a non-ICU level of care vs early ARDS at higher risk of decompensation would be very helpful to be able to appropriate triage the level of care and resource utilization.

The rates of COVID-19 positive UITs remain higher in both surge and non-surge periods compared to COVID-19 negative UIT patients; however, the difference between the two cohorts is less during the non-surge period. The higher mortality rates during the surge time can be multifactorial, including strained resource utilization and less knowledge and treatment effectiveness in the early stages of the disease process or the possibility of different lethality of different strains at different periods of disease. However, given the increased mortality of COVID-19 positive and COVID-19 negative patients during surge, it seems resource utilization and capacity have a larger impact than different strains or treatment modalities might, but further research is necessary to investigate.

The importance of trying to identify patients at risk for decompensation is again demonstrated to be of importance in this retrospective observation cohort. The patients described above illustrate a high rate of MV, longer LOS, and very high mortality rate among those who experienced ICU transfer within 24 hours. This is consistent with prior research in other disease pathologies that patient outcomes are poorly affected by unexpected deterioration after admission.

## LIMITATIONS

Given the research subject as it relates to time, all data was retrospective with some inherent limitations. One example is that there was no available matched population without UIT for comparison. Another limitation of our study is that our comparison groups were all during COVID-19, and practice patterns may have changed compared to practice prior to COVID-19. In addition, some of the COVID-19 negative patients may have been false negatives, which would have reduced the differences between the two groups. Furthermore, in making correlations there is no method to account for the different strains of COVID-19 at different time periods and their associated morbidity and mortality.

## CONCLUSION

COVID-19 has a higher rate of unexpected ICU transfer and higher mortality, which is anticipated and consistent with prior literature based on the disease progression in relation to respiratory failure. High inpatient volume times portends a higher risk of mortality for patients with unexpected ICU transfers within 24 hours of admission to a non-ICU level of care regardless of illness secondary to COVID-19 or otherwise.

## Figures and Tables

**Table 1:** Study population characteristics.

Data	Surge		Non-surge	
	
	COVID-19 positive (n = 60)	COVID-19 negative (n = 26)	COVID-19 positive (n = 24)	COVID-19 negative (n = 88)
Age (Median)	64.5	68.5	62.5	66.5
Gender (Male, n (%))	42 (70.0%)	18 (69.2%)	21 (87.5%)	49 (55.7%)
Race / Ethnicity (n (%))				
White	21 (35.0%)	17 (65.4%)	13 (54.2%)	50 (56.8%)
Black	7 (11.7%)	2 (7.7%)	1 (4.2%)	15 (17.0%)
Asian	4 (6.7%)	4 (15.4%)	4 (16.7%)	6 (6.8%)
Other	28 (46.7%)	3 (11.5%)	6 (22.2%)	17 (19.3%)
Not of Spanish/Hispanic origin	41 (68.3%)	24 (92.3%)	20 (83.3%)	72 (80.0%)
English-speaking (n (%))	35 (58.3%)	21 (80.1%)	14 (58.3%)	74 (84.1%)
Comorbidities (n (%))				
Hypertension	36 (69.0%)	18 (69.2%)	18 (75.0%)	50 (56.8%)
Hyperlipidemia	24 (40.0%)	15 (57.7%)	13 (54.2%)	36 (40.9%)
Diabetes	28 (46.7%)	6 (23.1%)	8 (33.3%)	30 (34.1%)
Coronary artery disease	11 (18.3%)	5 (19.2%)	5 (20.8%)	24 (27.2%)
Congestive heart failure	5 (8.3%)	3 (11.5%)	2 (8.3%)	20 (22.7%)
Asthma	7 (11.7%)	4 (15.3%)	1 (4.2%)	15 (17.0%)
Chronic obstructive pulmonary disease	4 (6.7%)	4 (15.3%)	1 (4.2%)	13 (14.8%)
Malignancy	5 (8.3%)	10 (38.5%)	4 (16.7%)	16 (18.2%)
Cirrhosis	0 (0%)	0 (0%)	0 (0%)	1 (1.1%)
Chronic kidney disease	10 (16.7%)	6 (23.1%)	3 (12.5%)	22 (27.2%)
End-stage renal disease on dialysis	5 (8.3%)	0 (0%)	0 (0%)	9 (10.2%)
Immunocompromised	9 (15.0%)	2 (7.7%)	0 (0%)	25 (29.4%)
Body mass index (average)	29.9	28.2	30.1	27.8
Smoking history (n (%))	9 (15.0%)	17 (65.4%)	12 (50.0%)	45 (51.1%)

*COVID-19*, coronavirus disease 2019.

**Table 2:** Description of therapies administered and patient outcomes.

Data	Surge		Non-surge	
	
	COVID-19 positive (n = 60)	COVID-19 negative (n = 26)	COVID-19 positive (n = 24)	COVID-19 negative (n = 88)
Maximum Oxygen Therapy in ED (n ([%])				
None	5 (8.3%)	10 (38.5%)	1 (4.2%)	49 (55.7%)
Nasal cannula	10 (16.7%)	9 (34.6%)	11 (45.8%)	25 (28.4%)
Facemask	21 (35.0%)	1 (3.9%)	0 (0%)	2 (2.3%)
High-flow nasal cannula	10 (16.7%)	1 (3.9%)	11 (45.8%)	4 (4.6%)
Non-invasive ventilation	5 (8.3%)	2 (7.7%)	1 (4.2%)	6 (6.8%)
Invasive mechanical ventilation	9 (15.0%)	3 (11.5%)	0 (0%)	2 (2.3%)
Time from admit to ICU transfer (average, hours)	13.0	10.3	12.8	11.5
Time from admit to death (average, days)	10.8	8.3	19.0	6.6
Oxygen therapy inpatient (n [%])				
High-flow nasal cannula	32 (53.3%)	8 (30.8%)	21 (87.5%)	10 (11.4%)
Non-invasive ventilation	16 (26.7%)	7 (26.9%)	13 (54.2%)	13 (14.8%)
Invasive mechanical ventilation	46 (76.7%)	5 (19.2%)	12 (50.0%)	14 (15.9%)
Total MV days (average, days)	13.6	5.4	8.1	5.2
Total ICU LOS (average, days)	11.9	4.7	8.8	3.7
Total hospital LOS (average, days)	16.6	7.7	14.5	8.2
Mortality (n [%])	38 (63.3%)	8 (30.8%)	9 (37.5%)	10 (11.1%)

*ED*, emergency department; *ICU*, intensive care unit; *MV*, mechanical ventilation; *LOS*, length of stay.
